# Evaluating the Role of Viable Cells, Heat-Killed Cells or Cell-Free Supernatants in Bacterial Biocontrol of Fungi: A Comparison Between Lactic Acid Bacteria and *Pseudomonas*

**DOI:** 10.3390/microorganisms13010105

**Published:** 2025-01-07

**Authors:** Francesca Di Rico, Francesco Vuolo, Edoardo Puglisi

**Affiliations:** 1 Department of Food Science and Technologies for Sustainable Agro-Food Chain (DiSTAS), Università Cattolica del Sacro Cuore, 29122 Piacenza, PC, Italy; francesca.dirico@unicatt.it (F.D.R.); edoardo.puglisi@unicatt.it (E.P.); 2 Sacco System, 22071 Cadorago, CO, Italy

**Keywords:** microbial biocontrol agents, plant pathogens, *Pseudomonas fluorescens*, *Lactiplantibacillus plantarum*, *Fusarium oxysporum*, cell-free supernatants, heat-killed cells

## Abstract

This study investigated whether viable cells, dead cells or cell-free supernatants (CFS) were responsible for the biocontrol effect of strains from two important bacterial genera, *Pseudomonas* and *Lactobacillus*, known for their antifungal properties against plant pathogens and food spoilage microorganisms. Specifically, the capability of these strains to produce extracellular hydrolytic enzymes on specified media was assessed, along with their effectiveness in inhibiting the mycelial growth of several phytopathogenic fungi (*Fusarium oxysporum*, *Botrytis cinerea*, *Pythium ultimum* and *Rhizoctonia solani*) using dual culture plate assays. Results from these inhibition assays revealed that *P. fluorescens* PF05 and *L. plantarum* LMG 23520 strains were the most effective in suppressing fungal growth, especially *F. oxysporum*. Therefore, further experiments were carried out to investigate the antifungal potential of the viable cells, heat-killed cells (HKC) and CFS from these strains against the germination of *F. oxysporum* spores. The viable cell trial proved successful, whereas HKC from the two bacterial isolates were ineffective against fungal spore germination. Conversely, the CFS of *L. plantarum* LMG 23520 was able to prevent fungal spore development for up to six days. The CFS of *P. fluorescens* PF05, instead, did not yield positive results. Additional studies are required to evaluate the potential inhibitory effects of the CFS from *P. fluorescens* PF05 and the HKC from both strains.

## 1. Introduction

Plant pathogens represent one of the greatest threats in the agricultural sector, as they can compromise the quality and productivity of crops by up to 30%, causing significant economic losses. Climate change, intensive agricultural practices and market globalization contribute to the spread of diseases, making plants increasingly susceptible to infections and exacerbating the reliance on chemical pesticides to control phytopathogens [1,2]. Synthetic products have long been employed to combat plant pests such as bacteria, fungi, viruses and insects but their intensive use can lead to pathogen resistance, environmental damage and risks to human health [3]. As a result, there is a growing trend towards more sustainable and eco-friendly disease management strategies, such as using microbial biocontrol agents (MBCA). These agents are beneficial microorganisms or their derivatives that can fight plant pathogens, offering a promising and reliable alternative to conventional pesticides [3,4].

MBCA are naturally found in the soil, especially in the rhizosphere, but they can also be present, though in smaller amounts, in the endosphere and phyllosphere [1]. The most well-known MBCA are found within genera such as *Pseudomonas* spp., *Bacillus* spp., *Burkholderia* spp., *Streptomyces* spp. and *Trichoderma* spp., which are effective against pathogens that cause foliar and soil-borne diseases, such as *Agrobacterium radiobacter* var. *radiobacter*, *Erwinia* spp., *Fusarium* spp., *Rhizoctonia solani*, *Phytophthora* spp. and *Pythium* spp. [5]. MBCA antagonize plant pathogens through several mechanisms, including competition for space and nutrients, hyperparasitism, secretion of hydrolytic enzymes, production of antimicrobial secondary metabolites like antibiotics, bacteriocins, siderophores and volatile compounds, and by inducing resistance in the host plants [4,5]. Special attention should be given to the genus *Pseudomonas*, with a focus on fluorescent pseudomonads, which are among the most well-characterized biocontrol agents. These bacteria are widespread in all plant environments and are effective in managing plant diseases due to their excellent ecological adaptability, powerful antagonistic action against a broad spectrum of pathogens and ability to stimulate an immune response in plants [1]. Moreover, many biocontrol agronomical products already use microbial inoculants based on *Pseudomonas*. In recent years, lactic acid bacteria (LAB) have gained increasing relevance as MBCA, owing to their potential in controlling a broad spectrum of bacterial and fungal phytopathogens [6]. These bacteria exhibit a wide range of diversity and adapt to various ecological niches, thriving in carbohydrate-rich environments such as milk and dairy products, plant materials (decomposing organs, leaves, rhizosphere, etc.), compost, silages, fermented foods and beverages, fermented fish and meat and even water [7]. LAB are promising candidates for biocontrol also for their well-established role in the biopreservation of many foods, protecting them from pathogenic and spoilage bacteria and fungi. Moreover, many of these strains are classified as Generally Recognized as Safe (GRAS) by the Food and Drug Administration (FDA) [8]. This study aimed to explore and compare the biocontrol potential of various *Pseudomonas* spp. and LAB by assessing (i) their ability to produce hydrolytic enzymes, (ii) their effectiveness in inhibiting the in vitro growth of key fungal phytopathogens and (iii) the impact of viable cells, HKC and CFS from the top-performing strains on the germination of *F. oxysporum* spores. The comparison between living cells, dead cells and supernatants has been conducted to evaluate the effectiveness of different formulations of the same microorganism for biocontrol against phytopathogenic fungi. Living cells play a crucial role as they utilize various modes of action, such as competition and hyperparasitism, while also adapting to their environment by producing antimicrobial metabolites in response to external factors, ensuring long-lasting biocontrol.

On the other hand, supernatants offer several advantages, including ease of ap-plication, immediate effects against pathogens and simpler production processes. However, they often require higher doses, which can pose environmental risks and contribute to the development of resistance in pathogens. Additionally, supernatants are more susceptible to contamination and can suffer from metabolite degradation over time, as they lack live cells [4].

The inclusion of HKC in this study aimed to evaluate their potential in biocontrol formulations. Although dead cells do not produce active metabolites, they may still exert antifungal effects through the release of extracellular compounds or the persistence of cellular structures that contribute to antagonism. Moreover, HKC provide advantages in terms of storage and handling, but their inability to produce active metabolites limits their effectiveness as biocontrol agents.

A key question in this study is whether there was a difference in antifungal activity between suspensions of dead cells and supernatants from which cells have been removed.

One hypothesis is that HKC might produce similar effects to CFS, offering a more cost-effective alternative by simplifying the production process. This could reduce the need for ongoing cell cultivation and complex procedures, making the overall biocontrol formulation easier and cheaper to produce.

## 2. Material and Methods

### 2.1. Origin of the Bacteria and Fungi

All bacterial and fungal strains used in this study were provided by the Italian company Sacco srl, Cadorago, Italy. The bacterial strains include several *Pseudomonas* and LAB species (*P. putida* PZY6, *P. putida* PSP1, *P. fluorescens* PF05, *P. protegens* PSPR04, *P. fluorescens* PF4.89, *L. plantarum* LMG 23520 and *L. paracasei* LMG 23518) isolated from the wheat rhizosphere and milk, respectively. The phytopathogenic fungi (*Fusarium oxysporum*, *Botrytis cinerea*, *Pythium ultimum* and *Rhizoctonia solani*) were obtained from diseased plants. The *Pseudomonas* and LAB strains were specifically chosen for their previously demonstrated pathogen defense features, making them suitable for further evaluation in biocontrol studies against key fungal phytopathogens.

### 2.2. Production of Extracellular Hydrolytic Enzymes

The ability of *Pseudomonas* and LAB strains to produce hydrolytic enzymes such as proteases, lipases, amylases, cellulases and chitinases was evaluated on Skim Milk agar, Nutrient agar supplemented with 5% of egg yolk, Nutrient agar supplemented with 1% starch and M9 supplemented with 1% carboxymethylcellulose and chitin-based medium (1%). These media were prepared in accordance with the protocols outlined by Herrera-Quiterio et al. and Bhattacharyya et al. [9,10]. In short, bacterial suspensions were spotted onto each medium and then incubated at 30 °C under aerobic conditions (for *Pseudomonas* strains) or at 37 °C under anaerobic conditions (for LAB strains) for different times depending on the type of medium. Enzymatic activity was indicated by the formation of a clear halo around the bacterial colonies.

### 2.3. Antifungal Activity Assays on Petri Plates

*Pseudomonas* and LAB strains were assessed for their ability to inhibit the growth of phytopathogenic fungi *F. oxysporum*, *B. cinerea*, *P. ultimum* and *R. solani* using the dual culture plate assay, based on Bellotti et al.’s protocol [11]. Briefly, 15-day-old mycelial discs (9 mm diameter) were cut from a vigorously growing margin of each fungal pathogen and placed at the center of a Petri dish containing PDA or modified MRS agar for fungi. Each bacterial suspension, standardized to a concentration of 3.00 × 10^8^ CFU/mL, was streaked parallel to and on both sides of the mycelial disc at 25 mm from it. Monoculture plates of each pathogen were used as negative controls. Mycelial growth was measured after 7 days of incubation at 25 °C by recording the fungal colonies’ area using ImageJ. The percentage of pathogen development inhibition compared to the control was calculated using the following formula:

Mycelial growth inhibition (%) = [(C − T)/C] × 100

where C = fungal colony area in the control group; T = fungal colony area in the treatment group.

### 2.4. Assessing the Antifungal Potential of P. fluorescens PF05 and L. plantarum LMG 23520 Against F. oxysporum Spore Germination

In the present study, the antifungal potential of the strains that performed best in the dual plate assays was assessed, focusing on viable cells, HKC and CFS from *P. fluorescens* PF05 and *L. plantarum* LMG 23520 against *F. oxysporum* spore germination. This investigation followed the protocols described by Bodil Kjeldgaard et al. [12], with some modifications. In all trials *P. fluorescens* PF05 was cultured in TSB and *L. plantarum* LMG 23520 in MRS broth. The viable cell experiment was conducted after overnight growth of the bacterial strains, with the cultures standardized to 10^8^ CFU/mL. For HKC and CFS assessments of *P. fluorescens* PF05 and *L. plantarum* LMG 23520, bacterial culture samples of both strains were collected at 14 and 24 h of incubation and quantified by measuring absorbance at 600 nm. These samples were then centrifuged to separate the supernatant from the bacterial pellet, which was autoclaved (120 °C for 15 min) to kill the cells. The supernatant was filtered to remove any cellular residues. Serial dilutions of viable cells, HKC and the CFS of *P. fluorescens* PF05 or *L. plantarum* LMG 23520 were co-inoculated with fungal spores of *F. oxysporum* (10^5^ spores/mL) in microtiter plates containing media suitable for fungal and bacterial growth (PDA, PDB, modified MRS agar or modified MRS broth for fungi). All plates were incubated at 25 °C and visually monitored for one week. For HKC and CFS tests in liquid media, absorbance at 600 nm was measured throughout the incubation period, alongside visual observations, and fungal growth inhibition was calculated using the following equation:

Inhibition of *F. oxysporum* spore germination (%) = [(Ac − At)/Ac] × 100

where Ac = absorbance (600 nm) in the control group; At = absorbance (600 nm) in the treatment group.

*F. oxysporum* spore suspension was prepared according to Parsa et al.’s protocol [13].

### 2.5. Statistical Analysis

All data were analyzed using one-way ANOVA followed by Tukey’s post hoc test (*p* < 0.05). Each experiment was conducted in triplicate to ensure the robustness of the results.

## 3. Results

### 3.1. Production of Extracellular Hydrolytic Enzymes

Enzyme activity assay results revealed that *P. fluorescens* PF05 strain exhibited the most pronounced protease activity (1.7 ± 0.10 cm), *P. putida* PZY6 showed the highest lipase activity (0.6 ± 0.1 cm), *P. putida* PSP1 was the top producer of amylase (0.33 ± 0.12 cm) and *P. protegens* PSPR04 demonstrated the highest cellulase production (1.23 ± 0.25 cm). Regarding the chitin-based medium, the *Pseudomonas* strains were able to grow on it, although no halo formation was observed around the colonies. In contrast, LAB, while showing protease activity on Skim Milk, particularly evident in *L. paracasei* LMG 23518 (0.50 cm), did not exhibit any other enzymatic activities.

### 3.2. Inhibition of Fungal Growth on Petri Plates

Table 1 presents data on the percentage inhibition of fungal growth of *F. oxysporum*, *B. cinerea*, *P. ultimum* and *R. solani* following treatment with *Pseudomonas* and LAB strains.

As shown, *P. fluorescens* PF05 exhibited the highest effectiveness against all four phytopathogenic fungi, particularly *F. oxysporum* (84.16%) and *B. cinerea* (78.45%). Other *Pseudomonas* strains moderately inhibited *F. oxysporum* (~35%) but had no impact on *B. cinerea*. Regarding *P. ultimum*, its growth was notably reduced by *P. fluorescens* PF05 (46.73%) and, to a lesser extent, by *P. protegens* PSPR04 (23.26%). The growth of *R. solani* was mainly inhibited by *P. fluorescens* PF05 (29.88%) and slightly by *P. fluorescens* PF4.89 (15.04%). *L. plantarum* LMG 23520 demonstrated the strongest inhibitory effect on *F. oxysporum* (41.52%) and weakly limited the proliferation of B. cinerea (14.85%). No significant differences were observed between *L. plantarum* LMG 23520 and *L. paracasei* LMG 23518 against *P. ultimum*. Regarding R. solani, the presence of LAB strains did not inhibit its growth; instead, it appeared to stimulate it.

### 3.3. Assessing the Antifungal Potential of P. fluorescens PF05 and L. plantarum LMG 23520 Against F. oxysporum Spore Germination

According to the inhibition assays, *P. fluorescens* PF05 and *L. plantarum* LMG 23520 were the best-performing in controlling fungal growth, especially that of *F. oxysporum*. The antifungal potential of viable cells, HKC and CFS from these two strains was then assessed to determine their ability to prevent the germination of *F. oxysporum* spores.

In the assay with viable cells, the antifungal activity of the *P. fluorescens* PF05 and *L. plantarum* LMG 23520 strains was evaluated and quantified by determining the minimum inhibitory concentration (MIC) required to stop the germination of *F. oxysporum* spores. For *P. fluorescens* PF05, mycelial growth inhibition was observed after just three days of incubation on PDA at a concentration of 10^8^ CFU/mL, and this effect persisted throughout the observation period. Similar results were obtained in PDB, although, in this medium, the MIC was only detectable after seven days. Additionally, the identified MIC aligned with the concentration that suppressed the growth of *F. oxysporum* in the dual plate assay. For *L. plantarum* LMG 23520, the MIC was 10^9^ CFU/mL in both modified solid and liquid MRS media. In the solid medium, inhibition was detected after just three days, while *F. oxysporum* took longer to grow in the liquid one. This test revealed that the concentration required to effectively hinder the germination of fungal spores was one log higher than what was needed to limit it in the inhibition assays.

**Figure 1:**
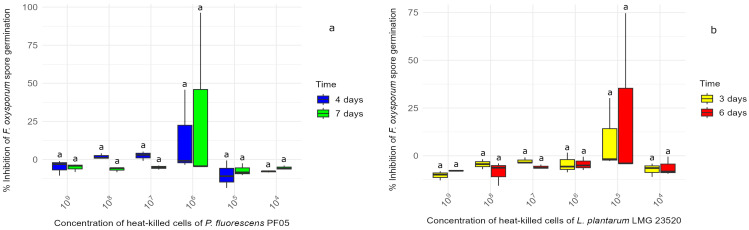
The box plots illustrating the percentage inhibition of *F. oxysporum* spore germination in liquid media following treatment with heat-killed cells (HKC) of (**a**) *P. fluorescens* PF05 and (**b**) *L. plantarum* LMG 23520, harvested and heat-killed after 24 h of incubation. Inhibition was measured at 4 and 7 days for *P. fluorescens* PF05 and at 3 and 6 days for *L. plantarum* LMG 23520. Statistical differences between HKC concentrations were determined separately for each time point using one-way ANOVA, followed by Tukey’s post hoc test (*p* < 0.05), with significant differences indicated by different letters.

In the experiment involving HKC of *P. fluorescens* PF05 and *L. plantarum* LMG 23520 collected at various time intervals, no suppression of mycelial growth was recorded on any type of medium. As shown in Figure 1, which depicts the results obtained in liquid media, none of the HKC concentrations from the 24 h incubation were capable of counteracting the development of *F. oxysporum* spores. In fact, most inhibition values were negative at both time points, and no statistically significant differences were observed in terms of efficacy between the concentrations at either 3/4 or 6/7 days. Similarly, the HKC of both strains, taken after 14 h, yielded comparable results.

**Figure 2:**
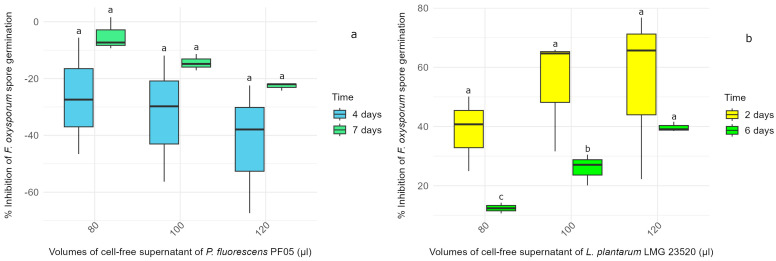
The box plots illustrating the percentage of inhibition of *F. oxysporum* spore germination in liquid media following treatment with varying volumes of cell-free supernatant (CFS) of (**a**) *P. fluorescens* PF05 and (**b**) *L. plantarum* LMG 23520, harvested after 24 h of incubation. Inhibition was measured at 4 and 7 days for *P. fluorescens* PF05 and at 2 and 6 days for *L. plantarum* LMG 23520. Statistical differences between CFS volumes were determined separately for each time point using one-way ANOVA, followed by Tukey’s post hoc test (*p* < 0.05), with significant differences indicated by different letters.

In the experiment using the CFS from *P. fluorescens* PF05 harvested at different times points, none of the tested volumes (80, 100 and 120 µL) were able to control spore germination. Mycelial growth was apparent from the start of incubation in both PDA and PDB media. As shown in Figure 2a, which presents the results from PDB, all data related to the inhibition of *F. oxysporum* germination treated with the supernatant of *P. fluorescens* PF05 collected after 24 h of incubation were negative. Similar results were observed with the CFS taken after 14 h.

The CFS from *L. plantarum* LMG 23520, collected after 24 h of incubation, effectively inhibited fungal spore germination in both solid and liquid media. In MRS agar, inhibition stopped after 3 days, while, in liquid medium, it persisted throughout the monitoring period. Indeed, as shown in Figure 2b, the inhibition results were promising after both 2 and 6 days, with higher volumes of supernatant producing stronger effects. Specifically, with 120 µL of CFS, inhibition reached 54.90% after 2 days and 39.72% after 6 days, showing a slight decrease but still remaining significant. To provide further insight, the pH of this CFS was measured and found to be 4. The CFS collected after 14 h, on the other hand, proved ineffective.

## 4. Discussion

In this study, various strains of *Pseudomonas* and LAB were evaluated as potential microbial biocontrol agents against fungal phytopathogens. The experiments assessed their ability to produce hydrolytic enzymes, reduce mycelial growth in vitro and examinate the antagonistic effects of viable cells, heat-killed cells and cell-free supernatants from the most promising strains on *F. oxysporum* spore germination. The emphasis on hydrolytic enzyme production was based on the rich literature and previous studies highlighting their effectiveness in breaking down fungal cell walls [14,15]. The results, however, revealed distinct patterns of enzymatic activity, with each *Pseudomonas* strain predominantly excelling in the production of one specific enzyme (protease, lipase, amylase or cellulase), suggesting a functional specialization. In contrast, LAB strains were limited to protease production, potentially reflecting their adaptation to environments where proteolytic activity is sufficient. This variability underscores the importance of considering strain-specific enzymatic profiles when selecting candidates for biocontrol applications. For chitinase activity, the *Pseudomonas* strains were able to grow on the chitin-based medium, indicating their potential to metabolize chitin. However, no clear halo formation around the colonies suggests that, although these strains may produce extracellular chitinase, its activity was not detectable with this method. In contrast, the LAB strains did not grow on the chitin-based medium, indicating a lack of chitinase activity in these strains.

In the dual plate assays, *P. fluorescens* PF05 and *L. plantarum* LMG 23520 demonstrated the greatest effectiveness in counteracting the growth of phytopathogens, particularly *F. oxysporum*. These findings align with extensive research demonstrating the inhibitory effects of *Pseudomonas* and LAB strains, both in vitro and in vivo [16,17]. An important mechanism by which *Pseudomonas* spp. combat pathogens is the production of bioactive compounds such as hydrogen cyanide, cyclic lipopeptides, phenazines, phloroglucinols, dialkylresorcinols, pioluteorin and pyoluteorin. These compounds are well documented for their antibacterial and antifungal properties [18]. Regarding LAB, their antimicrobial effect is mainly attributed to the production of organic acids such as lactic acid and acetic acid, as well as diacetyl, carbon dioxide, hydrogen peroxide, cyclic dipeptides, bacteriocins and other protein compounds [6,19].

The viable cells of *P. fluorescens* PF05 and *L. plantarum* LMG 23520 also succeeded in suppressing the germination of *F. oxysporum* spores, which exhibit greater resistance than the actively growing mycelium.

In the experiment involving HKC, cells from both strains were collected and killed after 14 and 24 h of incubation to evaluate potential differences in fungal suppression based on their growth stages. However, none of the HKC concentrations were successful in stopping fungal germination. This ineffectiveness could be attributed to the absence of active metabolite production in heat-killed cells, which limits their ability to directly inhibit fungal growth. Despite this, HKC may still have potential in biocontrol through indirect mechanisms, such as triggering plant defense responses in vivo. Further research could clarify their potential contribution to integrated plant protection strategies.

In the experiment with the CFS from *P. fluorescens* PF05, the supernatant taken at different growth stages was found to be ineffective in inhibiting both fungal growth and germination. This lack of efficacy may be attributed to the need for higher doses to achieve significant effect. Therefore, concentrating the supernatant through freeze-drying and retesting it could be a worthwhile approach.

The CFS from *L. plantarum* LMG 23520, extracted after 24 h of incubation, has shown a strong ability to hinder the germination of *F. oxysporum* spores. This effect likely results from the production of active antifungal secondary metabolites, which may increase as the bacterial growth progresses.

With a pH of 4, the CFS suggests that this inhibitory action may be due to the presence of acids like lactic acid. Additionally, the resulting fungal growth reduction was more persistent in liquid medium than in a solid one. This could be because, in a liquid environment, the compounds can diffuse more easily, creating a uniform setting that improves their interaction with *F. oxysporum* spores. Several studies have highlighted the antifungal efficacy of supernatants from various *Lactobacillus* strains against a range of fungal pathogens, supporting the findings of this investigation [20,21].

## 5. Conclusions

This study identified *P. fluorescens* PF05 and *L. plantarum* LMG 23520 as promising candidates for the biocontrol of fungal phytopathogens. These strains demonstrate significant antifungal activity, making them ideal candidates for development into beneficial microorganism-based formulations. Such formulations could be used to prevent or treat fungal diseases in agricultural systems, providing a sustainable alternative to chemical fungicides. Additionally, their antifungal properties hold potential for use in food preservation, where they could help extend shelf life by inhibiting fungal spoilage. To better understand the specific mechanisms behind the antifungal effects, further RNA-seq studies will be conducted, which will help identify key compounds and metabolic pathways responsible for their antagonistic activity. These findings will aid in optimizing the application of these strains in both agriculture and food industries.

## Figures and Tables

**Table 1:** Percentage of growth inhibition of *F. oxysporum*, *B. cinerea*, *P. ultimum* and *R. solani* by *Pseudomonas* and LAB strains. Statistical differences among bacterial strains for each fungal species, indicated by different letters, were assessed using one-way ANOVA followed by Tukey’s post hoc test (*p* < 0.05).

	% Inhibition of Mycelial Growth			
*Pseudomonas* spp.	*F. oxysporum*	*B. cinerea*	*P. ultimum*	*R. solani*
PZY6	34.83 ± 5.28 b	−3.34 ± 1.99 b	8.10 ± 1.55 c	−12.51 ± 13.62 b
PSP1	35.95 ± 4.95 b	−0.74 ± 3.44 b	−0.56 ± 1.57 d	−14.46 ± 8.54 b
PF05	84.16 ± 1.5 a	78.45 ± 4.01 a	46.73 ± 3.12 a	29.88 ± 18.00 a
PSPR04	35.30 ± 6.88 b	−3.20 ± 1.12 b	23.26 ± 5.02 b	−9.45 ± 22.41 b
PF4.89	32.07 ± 8.43 b	−3.43 ± 1.88 b	−0.18 ± 0.90 d	15.04 ± 20.98 a
LAB strains	*F. oxysporum*	*B. cinerea*	*P. ultimum*	*R. solani*
LMG 23520	41.52 ± 9.37 a	14.85 ± 4.28 a	35.15 ± 33.44 a	−18.91 ± 16.51 a
LMG 23518	31.16 ± 10.70 ab	1.68 ± 1.09 b	36.66 ± 25.80 a	−24.55 ± 8.47 a

## Data Availability

Data are contained within the article.

## Abbreviations

CFS	Cell-Free Supernatants
LAB	Lactic Acid Bacteria
HKC	Heat-Killed Cells
MIC	Minimum Inhibitory Concentration
PDA	Potato Dextrose Agar
PDB	Potato Dextrose Broth
